# First record of *Psorergatoides* Fain, 1959 (Acari, Cheyletoidea, Psorergatidae) for the Balkan Peninsula with description of the cutaneous lesions on the wing membrane of its hosts *Myotismyotis* (Borkhausen, 1797) and *Myotisblythii* (Tomes, 1857) (Chiroptera, Vespertilionidae)

**DOI:** 10.3897/BDJ.10.e89514

**Published:** 2022-07-20

**Authors:** Nia Toshkova, Boyan Zlatkov, Albena Fakirova, Violeta Zhelyazkova, Nikolay Simov

**Affiliations:** 1 National Museum of Natural History, Bulgarian Academy of Sciences, Sofia, Bulgaria National Museum of Natural History, Bulgarian Academy of Sciences Sofia Bulgaria; 2 Institute of Biodiversity and Ecosystem Research, Bulgarian Academy of Sciences, Sofia, Bulgaria Institute of Biodiversity and Ecosystem Research, Bulgarian Academy of Sciences Sofia Bulgaria; 3 Department of Pathology, Military Medical Academy, Sofia, Bulgaria Department of Pathology, Military Medical Academy Sofia Bulgaria; 4 Centre de recherche des Cordeleirs, Paris, France Centre de recherche des Cordeleirs Paris France

**Keywords:** skin mites, *Psorergatoides*, mouse-eared bats, Balkan Peninsula, bat wing health

## Abstract

Healthy wing membranes are essential for bats. They are critical for maintaining the water balance and, during hibernation, they protect the bat’s body from dehydration. Assessing the state of the membrane visually is an easy and effective way to monitor a bat’s health and discover abnormal structures and infections in wild bat populations. During pre- and post-hibernation surveys of bats’ wings, we identified the presence of skin mites, *Psorergatoideskerivoulae* (Fain, 1959). The parasite causes cutaneous lesions on the wing membranes of the greater moused-eared bat, *Myotismyotis* (Borkhausen, 1797) and the lesser moused-eared bat, *Myotisblythii* (Tomes, 1857). The lesser mouse-eared bat is a new host for this parasite. Our study is the first to describe the histopathology of the infection on the wings of the greater and lesser mouse-eared bats. To our knowledge, this is the southernmost record of this parasite and the first mention of the genus *Psorergatoides* for the Balkans.

## Introduction

In bats, the wing area takes up to 85% of the total body surface area (Reeder and Cowles 1951) and is essential for their ability to fly and for important physiological functions like thermo- and osmoregulation (Kluger and Heath 1970, Bakken and Kunz 1988, Bassett and Studier 1988, Thomson and Speakman 1999). The wing consists of two layers of epithelium, separated by a thin layer of blood and lymphatic vessels, delicate nerves, muscles and specialised connective tissues (Quay 1970, Makanya and Mortola 2007). Visual assessment of the health of the wing membrane has been a useful tool for bat biologists (Reichard and Kunz 2009, Francl et al. 2011), particularly since the discovery of the white-nose disease caused by the lethal dermato-pathogen *Pseudogymnoascusdestructans* (Lorch et al. 2011, Warnecke et al. 2013, Wibbelt et al. 2013) that affects the skin and the underlying connective tissue of bats during hibernation. Even with an unaided eye, the skin can be examined for the presence of pathogens and for abnormalities in the colouration, elasticity and obvious physical damage. Reichard and Kunz (2009) described the wing damage index (WDI) which is based on the degree of wing damage including scarring and associated lack of pigmentation, holes, tears and necrotic tissue. We used this approach for two Bulgarian bat populations in order to investigate the presence of strange-looking cutaneous lesions on the wings of *Myotismyotis* (Borkhausen, 1797) and *Myotisblythii* (Tomes, 1857). These were proven to be caused by skin mites, *Psorergatoideskerivoulae* Fain, 1959. The aim of our study was to describe the histopathology of the lesions caused by this parasite and present new data on its distribution.

## Material and Methods

### Sample collection

We conducted netting surveys in November 2020, April 2021, October 2021 and May 2022 at two karst caves in Bulgaria, Balabanova Dupka (43.134 N, 23.040 E) and Ivanova Voda (N41.894, E24.880), where we captured a total of 450 bat individuals. Both caves are cold (air temperature usually below 5℃) and are used by bats all year round. In Balabanova Dupka, around 2500 *Myotisblythii* hibernate (own data) and in Ivanova Voda, the hibernation colonies consist of several bat species including a mixed colony of *Myotismyotis/blythii* (max. count 5600 individuals: own data). The sites were typically netted for at least two nights. We identified the species of captured bats and we recorded sex, reproductive condition, age (Anthony 1988), mass (to the nearest 0.25 g; Pesola AG spring scales from various retailers) and length of the right forearm (to the nearest 0.5 mm). Each wing of each bat that we captured during this study was photographed and analysed for the presence of mite infection.

Normal skin and lesion samples were collected using a skin biopsy punch (ø = 3 mm). The average area of the sampled skin was about 7 mm^2^. We followed all ethical requirements for working with bats. The research was carried out under permit by the Bulgarian Biodiversity Act (No 830/19.09.2020).

### Sample processing

The samples were fixed and stored in neutral buffered formalin for a couple of months, washed two times with phosphate buffer with Triton™ X-100 (0.3%), embedded in Paraplast Plus® and sectioned at 5 μm and stained with haematoxylin and eosin (H&E). To isolate the mites, after sectioning of one of the blocks, the remaining half of the sample was dissolved with xylene and washed two times with the same solvent at 45°C, each step for 1 hour. The sample was washed with absolute ethanol three times for 30 min, then with 70% ethanol for 30 minutes and eventually transferred in a staining block with 5% ethanol serving as a dissection media. Under a stereomicroscope, the outer epidermal layers were removed with forceps and the mites were extracted with a 0.2 mm minuten needle. The mites were submerged in a drop of lactic acid on a slide and set on a hotplate at 45°C for 2–3 minutes to macerate the soft tissues, then mounted on temporary concave depression cavity slides with lactic acid (Baker 2005). The specimens were examined and photographed through an Amplival (Carl Zeiss Jena) under a compound microscope with an (EOS 2000D, Canon) camera attached in brightfield and phase contrast. To improve the focus, multiple images were stacked with Helicon Focus (Helicon Soft). The samples were preserved at the National Museum of Natural History, Sofia, Bulgaria.

## Results

**Figure 1.**
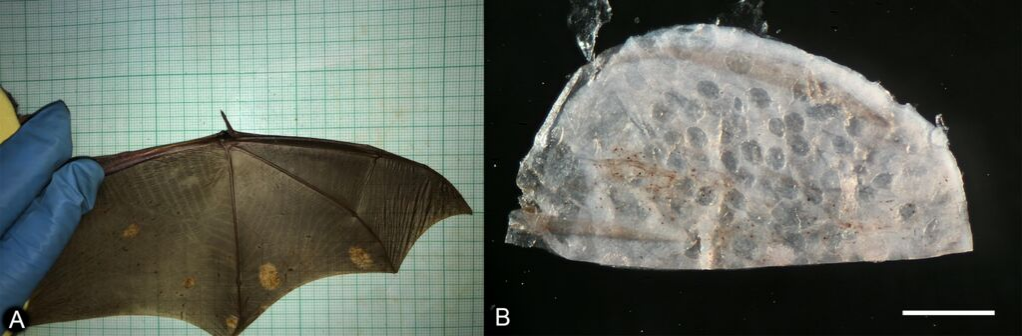
Wings of *Myotismyotis* infested with *Psorergatoideskerivoulae.* **A** Lesions manifested as pale-coloured spots on the wing membrane; **B** Dense infestation of mites was observed in the tissue samples. The darker ovoids are the holes remaining after the mites extraction. Scale bar 1 mm.

Over the course of the study, we collected information on the wing condition of 450 bat individuals from two study sites. We identified 16 individuals with lesions prior to the hibernation period, no individuals with signs of infection immediately after the hibernation period and three individuals with lesions one month after the beginning of the active season (end of May 2022). The shape of the lesions was mainly spherical and in some individuals (n = 10), there were multiple lesions on the same wing. The diameter of the lesions varied from 2 mm to 15 mm. Some of the severe cases were with multiple lesions on both wings covering up to 20% of the total wing surface area. Visually the lesions lack elasticity and resemble crumpled paper-like tissue with dark-orange colouration (Fig. 1). This makes it easy for field identification. We observed visual signs of infestation only on the wings of the study animals.

The microscopic examination of the bat wing sections with normal tissue morphology and with lesions showed the presence of parasitic mites in the latter. There are significant differences in histological characteristics between normal bat skin and the lesions. The bat wing membrane sections with usual morphology were covered with a single layer of cuboidal epithelium, with a focal intracellular brown pigment deposit. The subepithelial tissue was loose, with thin-walled vessels and some striated muscle fibres. In contrast, the degree of damage in some individuals suggested functional impairment of the derma. In the lesions’ sections, a focal transformation to squamous epithelium with superficial keratinisation was observed, around multiple ovoid vesicular structures intradermally, morphologically consistent with parasitic mites. Around the mites’ structures, there was an intense inflammatory infiltrate – lymphocytes, plasmocytes and some eosinophils and neutrophils. There were no muscle fibres observed (Fig. 3B-D).

**Figure 2.**
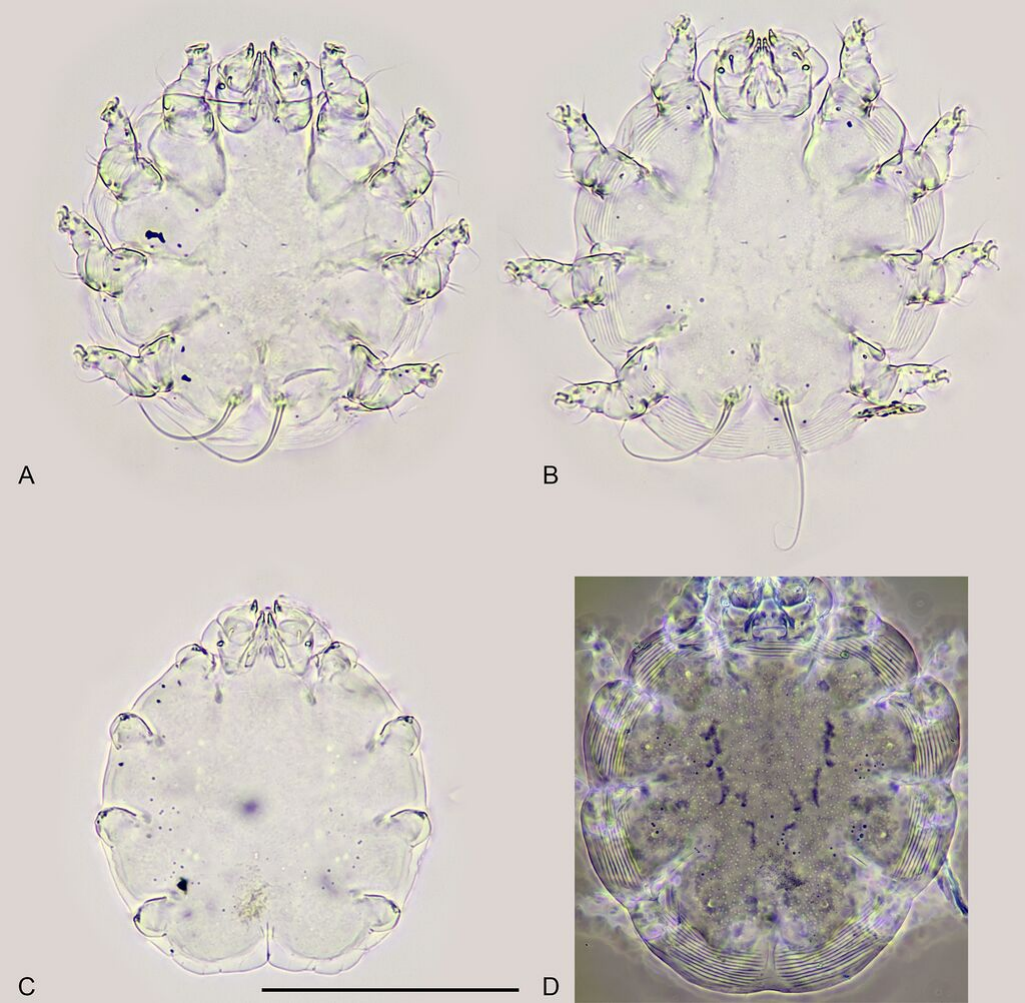
*Psorergatoideskerivoulae* from a wing of *Myotismyotis*. **A, B** females; **C** deutonymph; **D** female, dorsum of idiosoma (phase contrast). Scale bar 100 μm, all to scale.

**Figure 3.**
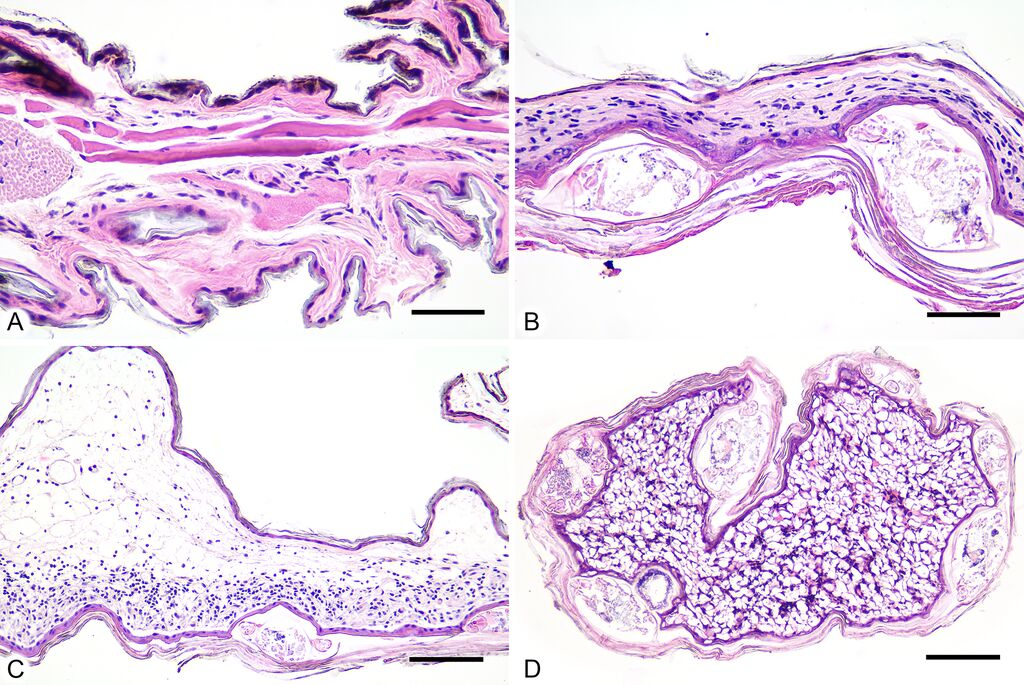
Histological sections of wing samples from *Myotisblythii b.* **A** Intact area; **B** Infested area with two *Psorergatoideskerivoulae* cross-sectioned; **C, D** Sections through acari-induced lesions with lymphoplasmacytic infiltrate. H&E staining. Scale bars: 50 μm (A, B), 100 μm (C, D).

The combination of the next features showed that the parasitic mites belong to the genus *Psorergatoides* Fain, 1959, the species *Psorergatoideskerivoulae* Fain, 1959: four pairs of strongly reduced dorsolateral shield setae, considered apomorphic of the genus (Fain 1959a, Fain 1959b, Nelson et al. 2017), two setae on femora I–III, only one seta on femur IV, length and width of dorsum ca. 120 μm, length of terminal setae more than 50 μm (Fig. 2).

Out of fifty specimens of *Psorergatoideskerivoulae* studied, both females (Figs. 2 A, B andD) and deutonymph (Fig. 2 C) were found. The density of the mite population was estimated to be 8 individuals/mm^2^. The mites were located very close to each other (Fig. 1B).

## Discussion

Skin mites are common in mammalian populations (Izdebska and Rolbiecki 2020) and over 20 families of various parasitic mites are associated with bats (Giesen 1990, Izdebska and Rolbiecki 2020). Representatives of the family Psorergatidae are small mono- or oligoxenic skin parasites and may cause skin lesions in the host (Giesen 1990, Izdebska and Krawczyk 2012, Nelson et al. 2017). Only a few literature sources are available about the clinical symptoms associated with mite infestation (Nelson et al. 2017). Although Psorergatidae seems to be common in the host populations (Izdebska and Fryderyk 2012), they are understudied because of their small size and practically asymptomatic presence in the skin in most cases (Izdebska and Fryderyk 2012, Nelson et al. 2017). As a whole, only two species are published for the fauna of the Balkan Peninsula and south-eastern Europe: *Psorergatesapodemi* Fain, Lukoschus & Hallmann, 1966 and *P.muricola* Fain, 1961 (Beron 2021). Our observation of *Psorergatoideskerivoulae* is the southernmost record for the species in Europe and the first mention of the genus *Psorergatoides* in the Balkans. *Psorergatoideskerivoulae* has an exceptionally wide geographic range and so far, it has been reported from Belgium, DR Congo, Ivory Coast, Malaysia (Borneo), Poland and Australia (Nelson et al. 2017, Beron 2021, Cierocka et al. 2022). The parasite has been associated with the following bat species: *Kerivoulacuprosa* Thomas, 1912, *K.lanosa* (Smith, 1847), *Myotisbocagei* (Peters, 1870), *M.macropus* (Gould, 1854), *M.muricola* (Gray, 1846), *M.myotis* (Borkhausen, 1797), *M.mystacinus* (Kuhl, 1817), *Plecotusauritus* (Linnaeus, 1758) (Chiroptera: Vespertilionidae) (Haitlinger 1979, Giesen 1990, Izdebska and Fryderyk 2012, Izdebska and Krawczyk 2012, Nelson et al. 2017, Beron 2021, Cierocka et al. 2022). The presence of mites in the skin lesions of the lesser mouse-eared bats (*Myotisblythii*) could be considered a new host-parasite association. The mechanisms and transmission pathways of Psorergatidae, usually show a narrow range of host specificity; thus the taxonomic status is questionable and undoubtedly will require future clarification.

The genus *Psorergatoides* infests Chiroptera hosts and often induces skin lesions and parasitosis which can rarely be lethal. The observed lesions in our studied individuals were relatively large. Some individuals were with multiple lesions with a diameter of around 10 mm. In comparison to other studies, the concentration of mites in our samples was higher (see Baker 2005). We observed infected individuals before hibernation (n = 16), no infected individuals immediately after the end of hibernation and three infected individuals one month after the beginning of the active season. There were evident infectious processes in those individuals and, to our knowledge, our study is the first to describe the histopathology of the infection. One explanation why we were not detecting any signs of mites infestation in the animals sampled directly after the end of hibernation can be related to the lifecycle of the parasites. Similarly to other bat skin mites, they might have reduced reproductive activity during the winter period due to the inactivity of the host and the low temperatures (Lourenço and Palmeirim 2008).

Our study sites are important bat hibernacula with records of the presence of the causative agent of the white-nose disease. A large fraction of the bats from the studied colonies exhibits visual signs of infection with *Pseudogymnoascusdestructans* (Zhelyazkova et al. 2020). Indeed, the skin mites infestation may be an additional stress factor for those populations and more research and conservation efforts are needed. Future research on the way hibernation affects the presence of these skin mites and how the infection affects the activity patterns during the winter season is needed. Another question open for discussion is whether the lesions lead to dehydration and disturbed osmoregulation, both crucial for the survival of the animals. New host-parasite association combined with wounded and damaged skin significantly increases the chance of other infections even with opportunistic pathogens (Robinson et al. 2019). In bats, a wide variety of commensal bacterial and fungal species are known to be enteropathogens and have the potential to cause opportunistic infections (Mühldorfer 2013).

Our study supports the validity of wing damage scoring as a cost-effective way to look at the general health of bats and as a useful tool for discovering pathogens. Damage to the wings does affect the foraging success of bats. Bats suffering from moderate wing damage are less manoeuvrable and have smaller foraging success, which indirectly leads to increased metabolic rate and can cause insufficient body fat storing (Voigt 2013). Lastly, some studies consider the connection between wing morphology and damage and the risk of extinction in bats (Jones et al. 2003, Safi and Kerth 2004). This further emphasised the need for more research efforts related to wing membrane diseases, damage causes and risk factors in bats.
